# Evaluation of *fli*C-*d* based direct blood PCR assays for typhoid diagnosis

**DOI:** 10.1186/s12866-016-0723-6

**Published:** 2016-06-13

**Authors:** Surojit Das, Ujjwayini Ray, Irfaan Akhter, Arka Chattopadhyay, Dilip Kumar Paul, Shanta Dutta

**Affiliations:** Bacteriology Division, National Institute of Cholera and Enteric Diseases, P-33, CIT Road, Scheme XM, Beliaghata, P.O. Box 177, Kolkata, 700010 West Bengal India; Microbiology Division, Apollo Gleneagles Hospitals, Kolkata, West Bengal India; Clinical Division, Dr. B. C. Roy Memorial Post Graduate Institute of Pediatric Sciences, Kolkata, West Bengal India; Department of Instrumentation and Electronics Engineering, Jadavpur University, Kolkata, West Bengal India

**Keywords:** Typhoid, Diagnostics, PCR, *fliC-d* gene

## Abstract

**Background:**

Typhoid cases need to be diagnosed accurately for early antibiotic therapy and reducing mortality. Identification of *Salmonella* Typhi (*S.* Typhi) in blood culture is conclusive, but has poor sensitivity. Detection of *S.* Typhi by PCR from blood sample has shown promise. Real-time quantitative PCR (Q-PCR) has been widely used in diagnostics for its rapidity and reliability. In the present study, the performance of molecular methods like conventional PCR (C-PCR), nested PCR (N-PCR) and Q-PCR were investigated and compared by targeting *S*. Typhi specific flagellar *fliC-d* gene directly in blood samples for typhoid diagnosis.

**Results:**

Analytical sensitivities and specificities of the PCR assays were determined under laboratory condition followed by diagnostic performances were demonstrated in 110 clinically diagnosed typhoid fever (CDTF) cases included as study subjects. The DNA detection limit of C-PCR was observed 3 × 10^4^ copies/reaction; those of N-PCR and Q-PCR (cutoff Ct value, ≤37) were 3 copies/reaction. The C-PCR was not further evaluated since it showed negative results with all clinical samples due to low sensitivity. Low isolation rate (21.8 %, 24/110) of *S*. Typhi by blood culture did not reflect the true burden of typhoid fever among the study subjects. Hence diagnostic performances of N-PCR and Q-PCR were determined considering CDTF cases positive by any of the diagnostic assay methods (*n* = 81) as true positives. Laboratory confirmed non-typhoidal cases (*n* = 29) were included as true negatives. On comparison, although both the assays were 100 % specific; sensitivity (91.4 % vs. 81.5 %) and efficiency (93.6 % vs. 86.4 %) of Q-PCR were better, but statistically not significant (*p* > 0.1) than N-PCR. The positive and negative likelihood ratios of Q-PCR were ∞ and 0.09 which indicated the potential clinical utility of Q-PCR for typhoid diagnosis. Q-PCR was more rapid than N-PCR (2 h vs. 6 h) in obtaining test results.

**Conclusions:**

This study demonstrates for the first time that TaqMan-based Q-PCR assay performs more favorably than N-PCR for direct detection of *S.* Typhi DNA in blood samples. Direct and quantitative blood Q-PCR is a rapid and reliable method for diagnosis of typhoid fever.

## Background

Accurate laboratory diagnosis of typhoid fever is mandatory for early antibiotic therapy, which reduces both mortality and morbidity. Around 11.9 million cases of typhoid fever with 129,000 deaths occur in low and middle income countries in 2010 [1]. This is probably an under estimate due to the lack of availability of suitable diagnostic test and absence of disease surveillance in developing countries. Clinical diagnosis of typhoid fever is also confusing due to the overlapping of symptoms with other febrile illnesses such as malaria, dengue, leptospirosis etc. [2].

Isolation of *Salmonella enterica* serovar Typhi (*S.* Typhi) by blood culture provides the most conclusive diagnosis of typhoid and is generally considered as the gold standard for validation of new diagnostic assays [3]. But since it suffers from poor sensitivity (40 to 60 %), use of blood culture as gold standard is challenging for evaluation of more sensitive molecular-based assays [4, 5]. Additionally microbiological culture requires 7 days for reporting negative result [2]. Culture of bone marrow aspirates is relatively more sensitive (>90 %) than blood culture but rarely practiced as the procedure is invasive [4, 6].

Over the last decade, *S*. Typhi DNA detection by direct blood PCR has shown most promising result for typhoid diagnosis. Of different genes (*fliC-d, hilA* and *viaB*) targeted in typhoid diagnostic PCR assays [7, 8], the flagellin gene (*fliC-d*, 1530 bp) of *S.* Typhi was commonly used due to its unique nucleotide sequences in hypervariable region VI of the gene which differ from those in other *Salmonella* serovars [9–13]. Use of nested PCR (N-PCR) and real-time quantitative PCR (Q-PCR) significantly improved the detection rate compared to that of conventional PCR (C-PCR) [9, 12, 14, 15]. But, these PCR diagnostic molecular methods could not be implemented into practice since issues like diagnostic utility of these techniques was neither demonstrated nor standardized under field situation in resource poor countries [12, 15].

Keeping the background information in mind, this study was undertaken to determine the performance abilities of *fliC-d* based direct PCR assays for typhoid diagnosis both under laboratory condition and field situation. Here we report the results on actual occurrence of the disease among hospital attending clinically diagnosed typhoid fever (CDTF) children in Kolkata by using C-PCR, N-PCR and TaqMan-based Q-PCR.

## Methods

### Bacterial strains

The bacterial strains used in this study are listed in Table 1, which consisted of both commercially available type strains as well as strains procured from bacterial repository of National Institute of Cholera and Enteric Diseases (NICED), Kolkata, India. The glycerol preserved stock strains, used for the study, were subcultured in LB (Luria-Bertani) broth or on LB agar (Difco, Sparks, MD) for extraction of DNA to be used as template in PCR assays.

**Table 1:** Bacterial strains used in this study

Sl. no.	Name of organism	Strain/sample ID	Result determined by		
			C-PCR	N-PCR	Q-PCR (Ct^a^)
1	*Salmonella* Typhi	MTCC 734	+	+	+ (19.1)
2	*Salmonella* Typhi	KOL 38	+	+	+ (20.0)
3	*Salmonella* Paratyphi A	KOL 24	-	-	- (40.0)
4	*Salmonella* Paratyphi A	MTCC 735	-	-	- (40.0)
5	*Salmonella* Typhimurium	NCTC 74	-	-	- (40.0)
6	*Salmonella* Typhimurium	BCH 7332	-	-	- (40.0)
7	*Salmonella* Enteritidis	BCH 7321	-	-	- (40.0)
8	*Salmonella* Enteritidis	EVS 111	-	-	- (40.0)
9	*Salmonella* Worthington	BCH 3008	-	-	- (37.0)
10	*Salmonella* Worthington	BCH 2770	-	-	- (40.0)
11	*Salmonella* Weltrevreden	OSS 56	-	-	- (38.5)
12	*Salmonella* Weltrevreden	OSS 57	-	-	- (40.0)
13	*Salmonella* Kentucky	EVS 318	-	-	- (40.0)
14	*Salmonella* Kentucky	EVS 319	-	-	- (40.0)
15	*Salmonella* Bareilly	EVS 44	-	-	- (36.9)
16	*Salmonella* Bareilly	EVS 45	-	-	- (39.1)
17	*Salmonella* Idikan	EVS 30	-	-	- (40.0)
18	*Salmonella* Idikan	EVS 31	-	-	- (38.4)
19	*Salmonella* Senftenberg	EVS 100	-	-	- (36.2)
20	*Salmonella* Virchow	EVS 160	-	-	- (36.7)
21	*Escherichia coli*	ATCC 35218	-	-	- (40.0)
22	*Escherichia coli*	U 1953	-	-	- (40.0)
23	*Escherichia coli*	U 2367	-	-	- (40.0)
24	*Escherichia coli*	U 2368	-	-	- (40.0)
25	*Escherichia coli*	BT 68	-	-	- (40.0)
26	*Escherichia coli*	BT 171	-	-	- (40.0)
27	*Klebsiella pneumonia*	U 1791	-	-	- (40.0)
28	*Klebsiella pneumonia*	U 1947	-	-	- (40.0)
29	*Klebsiella* sp.	P 1837	-	-	- (40.0)
30	*Klebsiella* sp.	P 1836	-	-	- (40.0)
31	*Klebsiella* sp.	P 1745	-	-	- (37.2)
32	*Acinetobacter* sp*.*	P 1872	-	-	- (40.0)
33	*Acinetobacter* sp.	JN 27	-	-	- (40.0)
34	*Acinetobacter* sp.	SP 2	-	-	- (38.2)
35	*Acinetobacter* sp.	BCR 154	-	-	- (40.0)
36	*Acinetobacter* sp.	BCR 188	-	-	- (40.0)
37	*Shigella flexneri* 2a	BCH 7286	-	-	- (38.2)
38	*Shigella dysenteriae*	BCH 5375	-	-	- (40.0)
39	*Shigella boydii*	BCH 4087	-	-	- (40.0)
40	*Shigella sonnei*	BCH 7178	-	-	- (40.0)
41	*Citrobacter freundii*	NTS 63	-	-	- (40.0)

C-PCR, conventional PCR; N-PCR, nested PCR; Q-PCR, real-time quantitative PCR; Ct, cycle threshold

^a^The Ct cutoff value for positive result is ≤30

### Study population

To assess the performances of direct PCRs for typhoid diagnosis under field situation, blood samples were collected from the study children of 2-12 years of age, attending the outpatient department (OPD) of Dr. B. C. Roy Memorial Post Graduate Institute of Pediatric Sciences, Kolkata, India in 2012. The children, who presented with high fever (>39 °C) for ≥3 days and clinically diagnosed as typhoid fever, were included as study subjects irrespective of history of antibiotic intake and severity/duration of the disease.

### Sample collection

Blood samples (5 ml) were collected aseptically from the study children and immediately inoculated into BactecPeds Plus bottles (Becton Dickinson, Bactec system, Franklin Lakes, NJ) for microbiological culture. Total DNA was extracted from 200 μl of citrated-blood samples from the study children using QIAamp DNA blood Mini Kit (Qiagen, Hilden, Germany) following manufacturer’s instruction. The extracted DNA was re-suspended in 200 μl of elution buffer for use as template in PCR assays.

### Ethical consideration

The present study was reviewed and approved by the Institutional Ethical Committee of NICED (Committee’s Reference ECR/416/Inst./WB/2013). Blood samples were collected from the febrile children after receiving written informed consent from their parents or guardians.

### Microbiological culture

The inoculated Bactec bottles were incubated at 37 °C for 7 days in Bactec 9120 system (Becton Dickinson) and subcultures were made on the MacConkey and nutrient agars (Difco, Sparks, MD) when the system showed alarm signal during the incubation period. Non-lactose fermenting smooth colonies was tested by Gram stains and other biochemical tests for *Salmonella* following standard protocol [16]. Confirmed identification of the *S*. Typhi was done by slide and tube agglutination using *Salmonella* O, H and Vi factor antisera (Denka Seiken Co Ltd., Tokyo, Japan). The result was read as negative if there was no growth after 7 days of incubation.

### Primers and probe used for PCR assays

For Q-PCR, the primers (qST-F, 5′-CTTGGCACAGGTTGATACACTT-3′; qST-R, 5′-GACATGTTGGAGACTTCGGTT-3′; amplicon size, 156 bp) and probe (qST-P, 5′-FAM-TGTCTTCTGCCCGTAGCCGTATCG-TAMRA-3′) were designed from the *fliC-d* gene of *S*. Typhi CT18 (GenBank accession number, AL513382) using Primer-Express Software (Applied Biosystems, Foster City, CA) and synthesized by outsourcing from Eurogentec (Seraing, Belgium). Similar primers have been used for C-PCR and second round of N-PCR to compare the analytical and diagnostic performances of all assays. Published primers (ST-1, 5′-ACTGCTAAAACCACTACT-3′; ST-2, 5′-TTAACGCAGTAAAGAGAG-3′; amplicon size, 462 bp) were used for the first round of N-PCR [9].

### PCR assays for detection of *S*. Typhi

#### C-PCR

The C-PCR was carried out in 25 μl volume comprising of 1x PCR buffer, 1U of *Taq* DNA polymerase, 250 μM of each dNTPs (New England Biolabs, Ipswich, MA), 0.3 μM of each primer (qST-F and qST-R) and 5 μl of template DNA. Amplification was carried out in a GeneAmp PCR System 9700 (Applied Biosystems) using the thermal condition of a pre-denaturation at 94 °C for 5 min, followed by 40 cycles of denaturation at 94 °C for 1 min, annealing at 60 °C for 1 min, extension at 72 °C for 1 min, and final extension at 72 °C for 7 min. Suitable positive and negative controls were used.

#### N-PCR

The first round of N-PCR was carried out using the PCR mix same as it was used for C-PCR, except the external primers (ST-1 and ST-2, 0.5 μM). The thermal cycling condition used was a pre-denaturation at 94 °C for 5 min, followed by 40 cycles of denaturation at 94 °C for 1 min, annealing at 52 °C for 1 min, extension at 72 °C for 1 min and final extension at 72 °C for 7 min. For the second round of N-PCR similar primers and thermal conditions were used like the C-PCR.

#### Q-PCR

The TaqMan-based Q-PCR was performed in 25 μl reaction mixtures containing 1x master mix (reaction buffer, FastStart *Taq* DNA polymerase, MgCl_2_ and dNTP [with dUTP instead of dTTP]) (Roche Diagnostics, Indianapolis), 0.3 μM of each primers (qST-F and qST-R), 0.1 μM probe (qST-P), 1x IPC mix (Exogenous internal positive control mix containing primers and Yakima Yellow-TAMRA probe), 1x IPC DNA (Eurogentec, Seraing, Belgium) and 5 μl of template DNA. Amplification was carried out in a StepOnePlus Real-Time PCR system (Applied Biosystems) using the pre-incubation at 95 °C for 10 min and two steps cycle (40 cycles) of denaturation at 95 °C for 15 s, annealing and extension at 60 °C for 1 min. The Universal Exogenous Q-PCR internal positive control (Eurogentec) was used to distinguish true target negatives and false negatives due to PCR inhibition, incorrect pipetting or erroneous cycling conditions.

### Sequencing of PCR products

The amplicons of three PCR assays were purified by QIAquick PCR purification kit (Qiagen) for direct sequencing using a 3730 DNA analyzer (Applied Biosystems), and analyzed by Basic Local Alignment Search Tool (BLAST) database search program of the National Center for Biotechnology Information (NCBI).

### Determination of analytical specificity and sensitivity

**Fig. 1:**
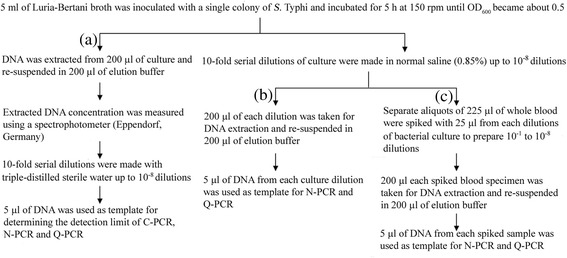
Flow chart showing methods for determination of analytical sensitivities of C-PCR, N-PCR and Q-PCR under laboratory condition. Three (**a**, **b**, **c**) categories of extracted DNA were used to determine the DNA detection limits. Bacterial culture showing one OD (at 600 nm) is equivalent to 8 × 10^8^ organisms/ml

Analytical specificities of the three PCR assays were determined by using genomic DNA of different bacterial strains (Table 1). Genomic DNA was extracted using the QIAamp DNA Mini Kit (Qiagen) following manufacturer’s instructions. The analytical sensitivities of the three PCRs were determined following the methods shown in Fig. 1. DNA concentrations were converted to DNA copy numbers using the formula; mol/g x molecules/mol = molecules/g via a DNA copy number calculator available at http://www.uri.edu/research/gsc/resources/cndna.html.

### Determination of diagnostic performances of PCR assays

Diagnostic performances of C-PCR, N-PCR and Q-PCR assays were evaluated using DNA templates extracted from citrated-blood samples of CDTF cases designated as study subjects.

### Statistical analysis

The sensitivities, specificities, positive predictive values (PPV), negative predictive values (NPV), positive likelihood ratio (LR+), negative likelihood ratio (LR-) and efficiencies of the PCR methods were calculated using the computer package SPSS for Windows (SPSS Benelux, Gorinchem, The Netherlands) considering CDTF cases positive by any of the diagnostic assay methods as true positives. The k-statistics was used to measure the agreement between N-PCR and Q-PCR assays as described previously [17]. McNemer (*χ*2) test and student’s *t*-test were used to determine the significance of difference between the test systems. A *p*-value of <0.05 was considered as statistically significant.

## Results

### Study subjects

A total of 110 hospital attending febrile children (65 males; 45 females) with CDTF were included in this study during 2012. Median age of the patient was 5 years (range, 7 months to 12 years) and median duration of fever was 8 days (range, 2 to 30 days). On enquiring to the accompanying parents, it was noted that 66 (60 %) cases had history of prior antibiotic intake, 19 (17.3 %) did not have any antibiotic, and 25 (22.7 %) were not sure about their status of antibiotic intakes.

Twenty-nine children (26.4 %) of 110 study subjects were included as negative controls. They were either laboratory confirmed non-typhoid cases (*n* = 20) or negative by all assay methods (*n* = 9). Among 20 non-typhoid cases, eight had dengue fever (positive by dengue IgM ELISA), five were positive for malaria parasites (*Plasmodium vivax*), seven had blood culture positive for bacteria other than *S.* Typhi, e.g, *S*. Paratyphi A (*n* = 2), *Acinetobacter* spp. (*n* = 2), *Pseudomonas* spp.(*n* = 2), *Klebsiella* spp. (*n* = 1).

### Determination of analytical specificity and sensitivity

Presence of visible bands (156 bp and 462 bp), confirmed by sequencing, was considered as positive results in the C-PCR and N-PCR assays. Both the assays yielded negative result for any bacteria other than *S*. Typhi (Table 1). In Q-PCR, the cycle threshold (Ct) value is the number of cycles required at a specific point when fluorescence rises prominently above the background noise. Low Ct values (Ct ≤20) were obtained in *S*. Typhi, which increased to >30 in other bacteria suggesting 100 % specificity of the Q-PCR assay (Table 1). For blood samples, when the Ct was >37, sequencing of the amplicons yielded dimers. In contrast, when Ct was ≤37, the amplicon sequences matched with *fliC-d* gene of *S*. Typhi indicating positive blood samples. Hence, we considered a cutoff Ct of ≤37 as positive result for blood samples.

**Fig. 2:**
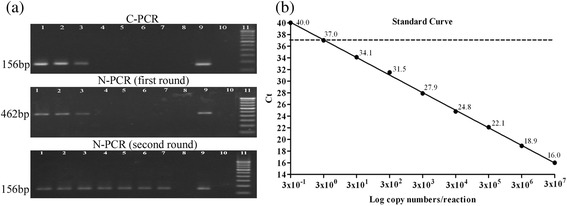
**a** Agarose gel (2 %) showing DNA detection limits of C-PCR and N-PCR by amplifying *fliC-d* gene for typhoid diagnosis. Lane 1 to lane 8, diluted bacterial DNA (10-fold) from 3 × 10^6^ to 3 × 10^−1^copies/reaction;lane 3, 3 × 10^4^copies/reaction (sensitivity of C-PCR); lane 7, 3 × 10^0^copies/reaction (sensitivity of N-PCR); lane 9, positive control (*S*. Typhi CT18); lane 10, negative control (sterile distilled water); lane 11, 100-bp DNA ladder marker. **b** Determination of analytical sensitivity of Q-PCR. A standard curve was generated by plotting the threshold cycles (Ct) values on *y* axis and DNA copy numbers/reaction on *x* axis. The dashed line indicates the Ct cutoff value of ≤37 at 3 × 10^0^ copies/reaction (sensitivity of Q-PCR)

DNA detection limits of the three PCR assays were determined using serial dilutions (10-fold) bacterial DNA from 1.2 × 10^5^ to 1.2 × 10^−3^ pg/reaction (corresponding to 3 × 10^7^ to 3 × 10^−1^ copies/reaction). The detection limit of C-PCR was found 120 pg/reaction (3 × 10^4^ copies/reaction), while N-PCR and Q-PCR assays could detect as few as 0.012 pg/reaction (3 copies/reaction; Ct = 37) (Fig. 2).

Since the detection limit of C-PCR assay was found 10^4^-fold higher than the other two PCR assays, and showed negative results for all clinical samples, C-PCR was not evaluated further for typhoid diagnosis. N-PCR and Q-PCR assays using categories (b) and (c) as DNA templates (Fig. 1) showed that detection limits of both the N-PCR and Q-PCR were 42 organisms/ml (Ct = 36.9), which corresponded to 10^−7^ dilution of *S.* Typhi culture (Table 2). In case of spiked blood samples, the detection limits of both N-PCR and Q-PCR were 420 organisms/ml (Ct = 36.5), which corresponded to 10^−6^ dilution (Table 2).

**Table 2:** Analytical sensitivities of N-PCR and Q-PCR using DNA extracted from different dilutions of *S.* Typhi cultures and spiked blood samples

Dilution factor	Organisms/ml	Result determined by			
		Bacterial culture		Spiked blood	
		N-PCR	Q-PCR (Ct^a^)	N-PCR	Q-PCR (Ct^a^)
Stock	4.2 × 10^8^	+	+ (16.0)	NA	NA
10^−1^	4.2 × 10^7^	+	+ (19.1)	+	+ (19.5)
10^−2^	4.2 × 10^6^	+	+ (22.5)	+	+ (23.1)
10^−3^	4.2 × 10^5^	+	+ (25.7)	+	+ (26.2)
10^−4^	4.2 × 10^4^	+	+ (28.2)	+	+ (29.8)
10^−5^	4.2 × 10^3^	+	+ (31.1)	+	+ (33.1)
10^−6^	4.2 × 10^2^	+	+ (33.6)	+	+ (36.5)
10^−7^	4.2 × 10^1^	+	+ (36.9)	-	- (39.2)
10^−8^	4.2 × 10^0^	-	- (40.0)	-	- (40.0)

NA, not applicable

^a^The Ct cutoff value for positive result is ≤37

### Determination of diagnostic performances of N-PCR and Q-PCR

Among 110 CDTF cases, Q-PCR showed higher sensitivity (91.4 %) in comparison to N-PCR (81.5 %) and culture (29.6 %) considering CDTF cases positive by any of the diagnostic assay methods as true positives (Table 3). The overall efficiencies of Q-PCR (93.6 %) and N-PCR (86.4 %) were significantly better than blood culture (48.2 %) (*p* < 0.0001), whereas no significant difference was found when the two PCR assays were compared (*p* > 0.1) (Table 3). Substantial agreement (k-value = 0.65) was observed between the two assays.

**Table 3:** Determination of diagnostic performance ability of blood culture, N-PCR and Q-PCR assays considering clinically diagnosed typhoid fever (CDTF) cases (*n* = 110) positive by any of the test methods as standard^a^

Tests	Sensitivity (%, 95 % CI)	Specificity (%, 95 % CI)	PPV (%, 95 % CI)	NPV (%, 95 % CI)	LR+(95 % CI)	LR-(95 % CI)	Efficiency (%)
Culture	24/81, 29.6 (20.0–40.8)	29/29, 100.0 (87.9–100.0)	24/24, 100.0 (85.6–100.0)	29/86, 33.7 (23.9–44.7)	29.6/0, ∞	70.4/100, 0.70 (0.61–0.81)	53/110, 48.2^b, c^
N-PCR	66/81, 81.5 (71.3–89.2)	29/29, 100.0 (87.9–100.0)	66/66, 100.0 (88.3–100.0)	28/44, 65.9 (49.0–79.0)	81.5/0, ∞	18.5/100, 0.19 (0.12–0.3)	95/110, 86.4^b, d^
Q-PCR	74/81, 91.4 (83.0–96.4)	29/29, 100.0 (84.4–100.0)	74/74, 100.0 (95.0–100.0)	29/36, 80.6 (64.0–91.8)	91.4/0, ∞	8.6/100, 0.09 (0.04–0.18)	103/110, 93.6^c, d^

CI, confidence interval; PPV, positive predictive value; NPV, negative predictive value; LR+, positive likelihood ratio; LR-, negative likelihood ratio

^a^Eighty one CDTF with any one test positive result as true positive and 29 laboratory-confirmed non-typhoid cases as negative controls

^b, c^ *p* < 0.0001 using McNemer test

^d^ *p* > 0.1 using McNemer test

**Fig. 3:**
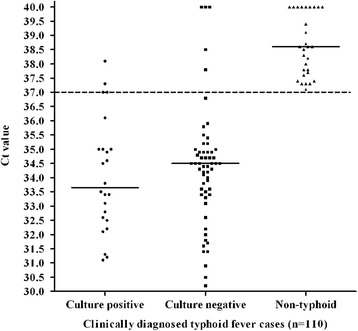
Distribution of Ct values by Q-PCR in blood culture positive typhoid (*n* = 24), culture negative typhoid (*n* = 57) and non-typhoid (*n* = 29) cases among the study subjects. The dashed line indicates the Ct cutoff value of ≤37 determining positive test by Q-PCR. The solid lines indicate the median Ct values for each group

The distributions of Ct values of 110 samples from CDTF cases were shown in Fig. 3. Among the 110 cases, 24 were culture positive, 57 were culture negative and 29 were non-typhoid cases. Median Ct values for culture positive, culture negative and non-typhoid cases were found 33.7 (range, 31.1 to 38.1), 34.5 (range, 30.2 to 40.0) and 38.6 (range, 37.1 to 40.0) respectively. Significant differences in the Ct distributions were found when culture positive and negative cases were compared with the non-typhoid cases (*p* < 0.0001), although the difference between culture positive and negative cases was not significant (*p* > 0.5). The standard curve (Fig. 2b) was used to calculate the bacterial DNA load among the study patients. The corresponding median bacterial DNA loads were calculated to be 5828 copies/ml (range, 193 to 42,960 copies/ml) and 3160 copies/ml (range, 0 to 85,654 copies/ml) in culture positive and negative samples respectively. The result of Q-PCR was available after 2 h, whereas it took 6 h for N-PCR.

## Discussion

Introduction of PCR into routine diagnostics has rapidly gained a pivotal role for diagnosis of a wide range of diseases, supplanting other conventional microbiological methods. This is true for typhoid fever also, where low bacterial count in the peripheral blood due to their intracellular existence in the reticulo endothelial system does not allow easy diagnosis by blood culture [2, 18]. Recently loop-mediated isothermal DNA amplification (LAMP) assay has been introduced for typhoid diagnosis which is still under investiagtion [19].

Among PCR assays, the Q-PCR has been well recognized to offer several advantages over N-PCR including quantification of bacterial load. When both the assays were compared, the Q-PCR was reported to show comparable or better result than N-PCR in many infectious diseases, but no such data was available for typhoid fever [20–22].

In this study, the analytical and diagnostic performances of Q-PCR and N-PCR assays targeting the *fliC-d* gene of *S*. Typhi were evaluated for typhoid diagnosis. Since same experimental conditions were used for both the methods, discrepancies in results could be attributed to the differences in detection limits of the respective method. In earlier reports, Ct values of ≤30 or ≤28 were regarded as positive for bacterial culture, and Ct values of ≤40 or ≤38 were positive for clinical samples [21, 23]. Similarly, based on the analytical assay results we have considered Ct values of ≤30 and ≤37 as positive for bacterial cultures and blood samples respectively.

The analytical specificities of the three PCR assays were found to be 100 % (Table 1). Analytical sensitivities of both N-PCR and Q-PCR assays were similar (3 copies/reaction) (Fig. 2). But, there was a 10-fold reduction in detection limit when spiked blood sample was compared with the bacterial culture in both N-PCR and Q-PCR (Table 2). Presence of human DNA or potential PCR inhibitors might be responsible for the decreased sensitivity of the spiked blood PCR. Detection limit of N-PCR was reported 0.04 pg (corresponding to 10 organisms) using blood DNA spiked with bacterial DNA as samples in earlier study [9]. Reported detection limits of Q-PCR were 1–5 DNA copies/reaction cloned in a plasmid vector and 250 organisms/ml in spiked blood samples [15].

Low isolation rate (21.8 %, 24/110) of *S*. Typhi among the study population indicated that blood culture did no longer reflect the true burden of the disease in a region and therefore should not be used as standard method. Hence we have used CDTF cases positive by any of the diagnostic assay methods as true positives and the sensitivity of Q-PCR (91.4 %) was found to be higher than N-PCR (81.5 %), but the difference was not statistically significant (*p* > 0.1). PCR-based molecular diagnostics has been adopted as gold standard not only for typhoid, but also for other infectious diseases [11, 24]. In this study, the LR+ and LR- of Q-PCR were ∞ and 0.09 which indicated the potential clinical utility of Q-PCR for typhoid diagnosis. Likelihood ratios (LRs) are used to measure and express diagnostic accuracy. LR+ value of ≥10 means that a positive test is good at ruling in a diagnosis, and LR- value of ≤0.1 indicates that a negative test is good at ruling out a diagnosis [25].

For typhoid diagnosis, *fliC-d-*based PCR assays has been evaluated by other researchers, who have reported ≥80 % sensitivity and 100 % specificity of N-PCR [9, 11, 12]. The sensitivity and specificity of Q-PCR were found 87 and 100 % in a study from Indonesia [14]. One study from Nepal showed limited sensitivity (42 %) of Q-PCR targeting a fimbrial-like adhesin gene of *S*. Typhi [15]. In this study, few culture confirmed typhoid fever cases showed negative results by N-PCR (*n* = 6) and Q-PCR (*n* = 2). Similar observation was also reported earlier by other researchers [9, 12, 15]. This may be due to the low number of bacteria in the patient’s blood below the detection limit of PCR assays.

Although higher copy number (median, 3160 copies/ml) of bacterial DNA was observed by Q-PCR assay in blood culture negative cases (*n* = 57), the higher rate (60 %) of antibiotic intake among the study population might lead to negative results in blood culture mehod (Fig. 3). In this study, the difference in DNA copy number obtained between blood culture positive and negative typhoid fever cases was not significant (*p* > 0.5); whereas one earlier study reported significant difference (*p* < 0.005) between the two categories (range, 1010 to 43,500 and 3.9 to 990 copies/ml) [14].

## Conclusions

This study demonstrates for the first time that TaqMan-based Q-PCR assay performs more favorably than N-PCR for direct detection of *S.* Typhi DNA in blood samples. Despite of relatively high cost of Q-PCR, it may be considered as the method of choice due to its rapidity, less chances of contamination, availability in a single-tube format and ability to obtain reproducible and quantitative results.

## Abbreviations

BLAST, Basic Local Alignment Search Tool; CDTF, clinically diagnosed typhoid fever; C-PCR, conventional PCR; Ct, cycle threshold; IPC, internal positive control; LAMP, loop-mediated isothermal DNA amplification; LB, Luria-Bertani; LR-, negative likelihood ratio; LR+, positive likelihood ratio; NCBI, National Center for Biotechnology Information; NICED, National Institute of Cholera and Enteric Diseases; N-PCR, nested PCR; NPV, negative predictive value; OPD, outpatient department; PPV, positive predictive value; Q-PCR: real-time quantitative PCR
